# The usefulness of casein-specific IgE and IgG4 antibodies in cow's milk allergic children

**DOI:** 10.1186/1476-7961-10-1

**Published:** 2012-01-02

**Authors:** Komei Ito, Masaki Futamura, Robert Movérare, Akira Tanaka, Tsutomu Kawabe, Tatsuo Sakamoto, Magnus P Borres

**Affiliations:** 1 Department of Allergy, Aichi Children's Health and Medical Center, Obu, Japan; 2 Division of Allergy, National Center for Child Health and Development, Tokyo, Japan; 3 Phadia AB (now Thermo Fisher Scientific), Uppsala, Sweden; 4 Department of Medical Sciences, Respiratory Medicine and Allergology, Uppsala University, Uppsala, Sweden; 5 Phadia KK (now Thermo Fisher Scientific), Tokyo, Japan; 6 Department of Medical Technology, Nagoya University School of Health Sciences, Nagoya, Japan; 7 Department of Hygiene, Yamaguchi University Graduate School of Medicine, Ube, Japan; 8 Department of Pediatrics, Sahlgrenska Academy of Göteborg University, Göteborg, Sweden

**Keywords:** casein, cow's milk allergy, IgE, IgG4, ImmunoCAP

## Abstract

**Background:**

Cow's milk allergy is one of the most common food allergies among younger children. We investigated IgE antibodies to milk, and IgE and IgG4 antibodies to casein, α-lactalbumin and β-lactoglobulin in cow's milk allergic (CMA) and non-allergic (non-CMA) children in order to study their clinical usefulness.

**Methods:**

Eighty-three children with suspected milk allergy (median age: 3.5 years, range: 0.8-15.8 years) were diagnosed as CMA (n = 61) or non-CMA (n = 22) based on an open milk challenge or convincing clinical history. Their serum concentrations of allergen-specific (s) IgE and IgG4 antibodies were measured using ImmunoCAP^®^. For the sIgG4 analysis, 28 atopic and 31 non-atopic control children were additionally included (all non-milk sensitized).

**Results:**

The CMA group had significantly higher levels of milk-, casein- and β-lactoglobulin-sIgE antibodies as compared to the non-CMA group. The casein test showed the best discriminating performance with a clinical decision point of 6.6 kU_A_/L corresponding to 100% specificity. All but one of the CMA children aged > 5 years had casein-sIgE levels > 6.6 kU_A_/L. The non-CMA group had significantly higher sIgG4 levels against all three milk allergens compared to the CMA group. This was most pronounced for casein-sIgG4 in non-CMA children without history of previous milk allergy. These children had significantly higher casein-sIgG4 levels compared to any other group, including the non-milk sensitized control children.

**Conclusions:**

High levels of casein-sIgE antibodies are strongly associated with milk allergy in children and might be associated with prolonged allergy. Elevated casein-sIgG4 levels in milk-sensitized individuals on normal diet indicate a modified Th2 response. However, the protective role of IgG4 antibodies in milk allergy is unclear.

## Background

Food allergies, described as adverse immune responses to food, are common and have increased in prevalence during the past decades. About 5% of the young children and 3-4% of the adults are affected today [1]. Milk, egg, peanut, tree nuts, fish, shellfish, wheat and soy are considered to cause most of the food adverse reactions [1]. Of these, cow's milk is the most frequent food causing allergy among infants and young children with a prevalence ranging from 1 to about 7.5% [2,3]. Proper management of milk allergy is important due to the low but serious risk of anaphylaxis [4]. Fortunately most children recover spontaneously from their allergy and develop tolerance to cow's milk until they reach 5 years of age [5,6]. The remaining children may have a prolonged cow's milk allergy causing discomfort and limitations to their daily lives for many years [7]. A recent study indicates that the proportion of children with prolonged milk allergy might be larger than previously anticipated [8].

The most important allergens in cow's milk are α-lactalbumin (also called Bos d 4), β-lactoglobulin (Bos d 5) and casein (Bos d 8) [2,9]. Milk can be separated into two fractions, the whey and the coagulum. Most known milk allergens are found in the milk whey including α-lactalbumin and β-lactoglobulin, while casein is present in the coagulum. Casein has been shown to be both more antigenic and allergenic than the whey proteins indicating its role as important milk allergen [10]. However, all milk proteins appear to be potential allergens and patients are often sensitized to several of them [11]. It has been shown that patients that are sensitized to several milk allergens tends to have a poor prognosis regarding outgrown of their milk allergy [12].

Diagnosis and management of food allergy include steps like initial avoidance of the suspected food allergen, skin prick testing and measuring of serum levels of food-specific IgE antibodies mostly using extract-based tests. In time, in order to confirm the diagnosis or to determine whether a reintroduction of the particular food is safe due to tolerance development, oral food challenges could be conducted [4]. Other diagnostic tools making it possible to earlier discriminate between prolonged and tolerated food allergies are desirable. We have earlier shown in wheat and egg allergy that specific IgE measurements could help the physician to provide better guidance to their patients and be a complement to food challenges [13,14].

Here, in order to study the clinical usefulness of specific antibodies in milk allergy, the concentrations of IgE antibodies to milk, and IgE and IgG4 antibodies to casein, α-lactalbumin and β-lactoglobulin were studied in sera from milk allergic and milk tolerant children.

## Methods

### Subjects

Eighty-three children with a suspected IgE-mediated cow's milk allergy (CMA) were enrolled in the study. The patients (male/female ratio, 55/28) ranged in age from 0.8 to 15.8 years (median: 3.5 years). All were milk sensitized as revealed by specific IgE *in vitro *test (n = 81), or had a history of positive skin prick test to milk (n = 2). Most of them suffered from atopic dermatitis (85%) and some also from asthma (32%). The patients were divided into two groups on the basis of their immediate reactions to an open oral milk provocation challenge test or through their case history. The cow's milk allergic (CMA) group (n = 61) had either a positive challenge result for milk (n = 34) or a convincing history of present milk allergy making a challenge test redundant (n = 27). The non-CMA group (n = 22) contained children who had no allergic reactions to milk at the time of examination. A subgroup of them had obtained tolerance after a previous diagnosed milk allergy (Tolerant, n = 11) which was verified with a negative challenge test with milk. The remaining children had never had a milk allergy diagnosis (Negative, n = 11). Further details about the study groups are found in Table 1.

**Table 1:** Patient characteristics of cow's milk allergic (CMA) children, diagnosed by food challenge (Challenge) or a convincing history of present milk allergy (History), and non-CMA children sensitized to milk.

	CMA (n = 61)		non-CMA (n = 22)	
	
	Challenge	History	Tolerant	Negative
Sex (boys/girls)	21/13	18/9	7/4	9/2
Age, years (median, range)	3.9 (1.0-12.7)	3.0 (0.8-12.8)	2.5 (1.0-15.8)	3.2 (1.4-14.2)
Serology (median, range)				
Total IgE (kU/L)	395 (32.0-12,784)	536 (34.0-14,283)	267 (44.0-3,260)	958 (66.1-27,815)
Milk-sIgE (kU_A_/L)	11.6 (< 0.35- > 100)	15.9 (1.4- > 100)	4.2 (< 0.35-27.6)	3.9 (1.0-17.3)
Casein-sIgE (kU_A_/L)	13.0 (< 0.35- > 100)	21.6 (0.42- > 100)	2.3 (< 0.35-6.6)	2.0 (1.1-4.2)
Allergic symptoms, no (%)				
Atopic dermatitis	28 (82%)	24 (89%)	9 (82%)	10 (91%)
Asthma	9 (26%)	12 (44%)	5 (45%)	1 (9%)
Egg allergy	25 (74%)	20 (74%)	4 (36%)	6 (54%)
Medication, no (%)				
H1-antagonist	6 (18%)	6 (22%)	3 (27%)	3 (27%)
Inhaled corticosteroids	2 (6%)	5 (18%)	-	-

The latter group consisted of a subgroup of children that had obtained tolerance after a previous diagnosis of milk allergy (Tolerant) and subgroup of children that never had had a milk allergy diagnosis (Negative).

For details see method section.

For the specific IgG_4 _analyses, a non-milk sensitized control group was included consisting of children that consulted our allergy clinic due to eczema, urticaria, bronchial asthma or any suspicion of allergic diseases and were examined for the following specific IgE antibodies; milk, *Dermatophagoides pteronyssinus*, cat dander, egg white, wheat, orchard grass, and Japanese cedar. Those who had no clinical history of milk allergy and without milk-specific IgE antibodies (< 0.35 kU_A_/L) were selected, and subsequently divided into the following two subgroups. One subgroup consisted of 31 non-atopic controls (NAC; median age: 1.0 years, range: 0.8-6 years). They had low total IgE levels (median: 13.0 kU/L, range: 2.0-29.0 kU/L) and were negative to all of the specific IgE antibodies examined. The other subgroup consisted of 28 atopic controls (AC; median age: 5.0 years, range: 1-15 years). They were sensitized to at least one of the allergens described above, except milk, and had a median total IgE level of 928 kU/L (range: 254-4,618 kU/L).

Informed consent was obtained from patients, their parents, or both. The study was approved by the Ethics Committee of Fujita Health University School of Medicine.

### Oral milk challenge

Open oral milk provocation tests were carried out as described in a Japanese guideline [15]. The children were orally challenged with raw milk, starting with one drop and followed with increasing volumes (1, 2, 5, 10, 20 or 30 ml) every 20 minutes, until a reaction was observed. Only objective immediate reactions were considered as positive results and the patients were given relevant relief medication thereafter. All provocation tests were conducted by qualified medical personnel.

### Serological analysis

Serum levels of total IgE, IgE antibodies to milk, casein, α-lactalbumin and β-lactoglobulin, and IgG4 antibodies to casein, α-lactalbumin and β-lactoglobulin were measured using ImmunoCAP^® ^(Phadia AB, Uppsala, Sweden). The commercially available tests and reagents were used according to the instructions from the manufacturer.

### Statistical analysis

The Mann-Whitney U-test (two-tailed) was used for comparisons between the groups and p values < 0.05 were considered significant. Receiver operating characteristic (ROC) analysis was performed for the different milk allergen ImmunoCAP tests used for specific IgE measurement [16]. Before statistical evaluation, all specific IgE values below the assay cut off (0.35 kU_A_/L) were assigned a value of 0.34 kU_A_/L, and all values above 100 kU_A_/L (higher limit of quantitation) were assigned a value of 101 kU_A_/L. In the same way, all specific IgG4 levels below the assay cut off (0.07 mg_A_/L) were assigned a value of 0.06 mg_A_/L.

## Results

### Milk allergen-specific IgE antibodies

**Figure 1:**
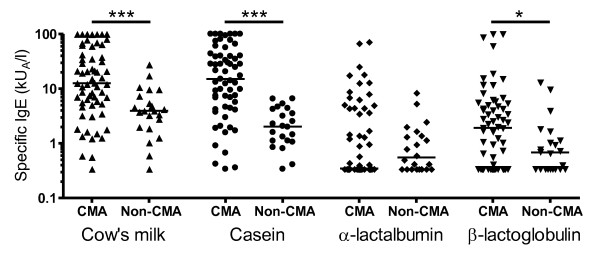
**Serum concentration of milk allergen-specific IgE antibodies in children with cow's milk allergy (CMA) and with tolerance to milk (non-CMA)**. The median levels are shown as indicated. * p < 0.05, *** p < 0.001.

No significant differences in total IgE levels between the CMA and non-CMA groups were seen (Table 1), while the CMA group had significant higher levels of milk-specific IgE antibodies as compared to the non-CMA group (Figure 1). Two children were negative for milk-specific serum IgE antibodies (< 0.35 kU_A_/L), but had a history of positive skin prick tests with wheal diameters of 12 × 4 and 15 × 9 mm, respectively.

The CMA patients had increased levels of casein-specific IgE antibodies as compared to the non-CMA patients. All but one CMA patient had casein-specific IgE > 0.35 kU_A_/L resulting in a 98% sensitivity for the casein ImmunoCAP test using the traditional assay cut off. The levels of β-lactoglobulin-specific IgE were also increased in the CMA group, but no differences between the groups were seen for α-lactalbumin-specific IgE. There were 19 patients without measurable β-lactoglobulin-specific IgE (< 0.35 kU_A_/L) and 30 patients without α-lactalbumin-specific IgE, resulting in clinical sensitivity values of 69% for the β-lactoglobulin test and 51% for the α-lactalbumin test, respectively (Figure 1).

**Figure 2:**
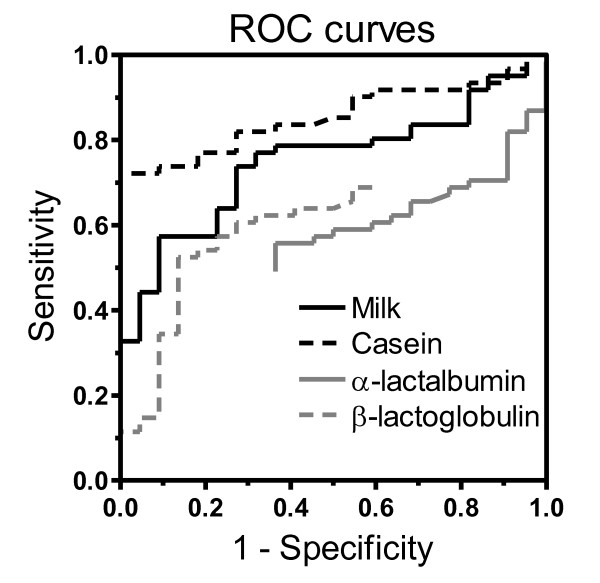
**ROC curves showing the performance of the ImmunoCAP tests for milk-, casein-, α-lactalbumin- and β-lactoglobulin-specific IgE antibodies in relation to the diagnosis of cow's milk allergy**.

The ROC analysis showed that the casein ImmunoCAP test was superior in its diagnostic performance compared to the milk, α-lactalbumin and β-lactoglobulin tests (Figure 2). It was especially outstanding in its clinical specificity. The ROC analysis for the casein ImmunoCAP test showed that, when using a clinical decision point corresponding to 6.6 kU_A_/L of casein-specific IgE, a specificity of 100% and a sensitivity of 72% could be achieved. At corresponding cut-off points with 100% clinical specificity, the milk and β-lactoglobulin tests showed clinical sensitivity values of just 33% and 11%, respectively. Using the α-lactalbumin ImmunoCAP test, a specificity of no better than 63% could be achieved in this patient material. No children with casein-specific IgE below the clinical decision point of 6.6 kU_A_/L had high levels of IgE antibodies to milk, β-lactoglobulin or α-lactalbumin.

**Figure 3:**
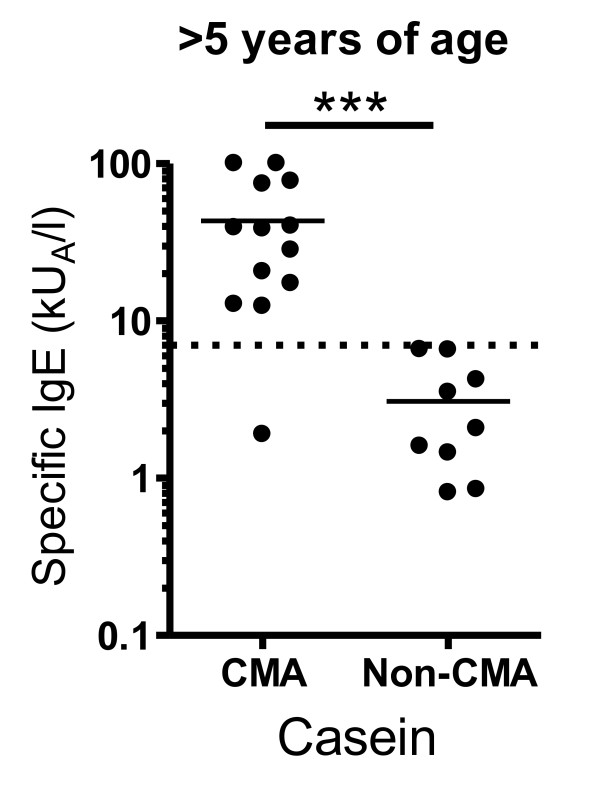
**Serum concentrations of casein-specific IgE antibodies in children aged > 5 years with cow's milk allergy (CMA) and with tolerance to milk (non-CMA)**. The median levels are shown as indicated. *** p < 0.001.

Tolerance for cow's milk is expected to be obtained until the age of five years for most allergic children [5,6]. When looking at the subgroup of children over five years of age, all but one of the CMA children had casein-specific IgE concentrations above our suggested positive prediction point of 6.6 kU_A_/L, while all of the older non-CMA children had lower levels (Figure 3). Also the IgE levels to milk and β-lactoglobulin were significantly elevated in CMA children compared to non-CMA children over five years of age, although not as prominent as for the casein-specific IgE (data not shown).

### Milk allergen-specific IgG4 antibodies

The serum levels of casein-, α-lactalbumin- and β-lactoglobulin-specific IgG4 antibodies were measured in the CMA and non-CMA groups as well as in a non-milk sensitized control group consisting of children with (AC subgroup) and without atopy (NAC subgroup) as defined by their sensitization to common inhalant and/or food allergens.

The non-CMA group had significantly increased levels of specific IgG4 antibodies to all three milk allergens as compared to the CMA group (p < 0.01 and < 0.001). However, when studying the Tolerant and Negative subgroups of the non-CMA group individually, it was shown that the increased levels of specific IgG4 were seen mainly in the Negative subgroup composed of children without previous history of milk allergy (Table 2).

**Table 2:** Levels of milk allergen-specific IgG4 antibodies.

Test	CMA group	Non-CMA group		Non-milk sensitized control group	
		
		Tolerant	Negative	AC	NAC
	n = 61	n = 11	n = 11	n = 28	n = 31
Casein	0.36 (< 0.07-13.4)	0.46 (< 0.07-25.4)	24.8 (0.17-197)	5.2 (0.14-64.7)	1.3 (< 0.07-125)
p-value	-	NS	< 0.001	< 0.001	NS
					
α-lactalbumin	< 0.07 (< 0.07-2.7)	< 0.07 (< 0.07-1.8)	7.1 (0.09-52.9)	2.0 (< 0.07-44.7)	1.2 (< 0.07-25.1)
p-value	-	NS	< 0.001	< 0.001	< 0.01
					
β-lactoglobulin	< 0.07 (< 0.07-3.5)	0.09 (< 0.07-1.8)	5.2 (< 0.07-16.5)	1.5 (< 0.07-20.6)	0.53 (< 0.07-28.2)
p-value	-	NS	< 0.001	< 0.001	< 0.001

Results are presented as median concentrations (mg_A_/L) with range.

For details about the different groups/subgroups, see method section. Significant differences compared to the CMA group are shown by the p-value. NS indicates non-significant difference.

Increased levels of specific IgG4 to α-lactalbumin- and β-lactoglobulin were also seen in the non-milk sensitized control group. However, increased levels of casein-specific IgG4 antibodies, as compared to the CMA group, were only found in the AC subgroup (Table 2). The overall highest levels of casein-specific IgG4 were observed in the Negative subgroup of children belonging to the non-CMA group followed in rank by the AC subgroup (p < 0.05) and the NAC subgroup (p < 0.001). The casein-specific IgG4 levels in the NAC subgroup were similar as in the CMA group and the Tolerant subgroup.

## Discussion

In the present study, it was shown that the levels of IgE antibodies to milk, casein and β-lactoglobulin were increased in Japanese children with milk allergy compared to milk-sensitized children without present symptoms to milk (non-CMA children). When comparing different ImmunoCAP tests for measurement of IgE antibodies to milk, casein, α-lactalbumin and β-lactoglobulin, the best performance in the diagnosis of milk allergy was shown for the casein test. All but one of the CMA children had casein-specific IgE antibodies (> 0.35 kU_A_/L). A majority of them (72%) had casein-specific IgE antibodies above our suggested clinical decision point of 6.6 kU_A_/L, while all non-CMA children were below this cut-off point for casein-specific IgE.

Milk allergy is an obvious health problem and burden for the individual allergic child. But the disease also causes limitations to the daily life of the immediate family in their effort of trying to avoid accidental exposure of their child to milk. A recent study showed that allergic reactions to accidental exposure to milk allergens indeed are frequent [17]. Avoiding casein and other cow's milk allergens might not always be so simple or feasible considering that all kinds of foods and dairy products may not have the proper labeling of the contents. Allergic reactions have also been reported as a result of casein being added as an extender to non-dairy products such as sausages and soups [18].

Double-blind, placebo-controlled food challenge is considered to be the gold standard for food allergy diagnosis, although it carries some risks for the patient and is time-consuming to conduct [15]. For several reasons many children are diagnosed as having a food allergy even though no food challenges are performed [4]. This may lead to an over-diagnosis that is very costly for the society. Even more important, it is negatively influencing the quality of life for the whole family that is concerned [19]. Serious food allergies may also be missed. Therefore, improved diagnostic tools including better knowledge how to interpret test results are crucial. For several years studies have been performed by leading allergologists to find clinical decision points for the diagnosis of food allergy using allergen-specific serum IgE measurements. Cut-off points for clinical milk allergy in 2-3 year old children have been reported by others to be 24 kU_A_/L for IgE antibodies to milk and 9 kU_A_/L for IgE to casein, respectively [20]. Thus, a similar decision point for the casein-specific IgE as found in the present study with Japanese children with a median age of 3.5 years. However, clinical decision points often varies between studies which can be explained by differences between the study populations and the statistical criteria for choosing the decision points [21]. For example, younger children generally have lower levels of IgE antibodies to milk compared to older children [20,22], something that has to be acknowledged when interpreting specific IgE test results in the diagnosis of children.

Studies have shown that milk-specific IgE levels are lower in children who later become tolerant than in those with prolonged allergy, showing that IgE antibody measurements can be used to predict tolerance development [8,23,24]. In our study, the levels of specific IgE to milk, casein and β-lactoglobulin were elevated in patients diagnosed with prolonged milk allergy (defined as having milk allergy at age > 5 years) as compared to milk-tolerant patients at similar age. Again, the casein test showed an excellent diagnostic performance, since all but one of the CMA children had casein-specific IgE levels above our suggested positive prediction point of 6.6 kU_A_/L. Also others have shown that patients with prolonged milk allergy generally have higher levels of milk-specific IgE than patients whose allergy has resolved [7,8], and the association between casein-specific IgE and prolonged milk allergy has been shown both in children [20,23] and in adults [25].

The production of IgG4 antibodies is considered to be a normal physiological response to the ingestion of cow's milk [26]. Previous studies have shown that individuals that tolerate cow's milk have higher levels of milk-specific IgG4 antibodies than those with a prolonged milk allergy [27,28], and it has been indicated that IgE and IgG4 antibodies combined might be used to predict tolerance development [29]. However, in our study the subgroup of non-CMA children who had obtained milk tolerance as diagnosed by challenge (Tolerant group) did not have significantly higher levels of casein-, α-lactalbumin- or β-lactoglobulin-specific IgG4 antibodies as compared to the CMA group, probably a consequence of their milk avoidance. Instead, the subgroup of children who never had had milk allergy, although sensitized to milk (Negative subgroup), showed elevated concentrations of IgG4 antibodies. This indicates that high levels of milk allergen-specific IgG4 antibodies are merely a reflection of a child's diet where cow's milk normally is included. So in line with the common opinion [30], our study supports that measurement of IgG4 antibodies has currently no role in the diagnosis of food allergy.

Interestingly, the Negative subgroup had the highest levels of casein-specific IgG4 of all groups in the study including the AC group and NAC group with non-milk sensitized children. It suggests that casein-specific IgG4 antibodies might be markers of a so-called modified Th2 response as suggested by Platts-Mills [31]. Thus, the milk allergen-specific IgG4 antibodies might have a protective role against the development of milk allergy, perhaps by blocking the antigen-presentation to allergen-specific T cells as described by others [32].

## Conclusions

IgE antibody levels to milk, casein and β-lactoglobulin were increased in Japanese children with cow's milk allergy. The casein ImmunoCAP test showed the best clinical performance. A majority of the CMA children had casein-specific IgE levels above our suggested positive decision point of 6.6 kU_A_/L, while all non-CMA children were below this cut-off point. It was also shown that the levels of casein-specific IgE remained high in CMA children also after five years of age. We therefore conclude that high levels of casein-specific IgE antibodies are strongly associated with milk allergy in children and might be associated with a prolonged allergy. Results from the present study indicate that high levels of casein-specific IgG4 antibodies are associated with tolerance in milk-sensitized children, but only in subjects on normal milk-containing diet. The protective role of IgG4 antibodies in a so-called modified Th2 response is unclear, and it has not yet been proven that measurement of IgG4 antibodies has a role in the clinical management of food allergy. Thus, more studies are needed before proposing routine use of specific IgG4 measurements in milk allergy.

## Competing interests

The authors declare that they have no competing interests.

## Authors' contributions

All authors have contributed to the final interpretation of data and the writing of the manuscript that has been approved by all parts. Furthermore, KI initiated and coordinated the Japanese part of the study. MF carried out sample collection. RM reviewed the results and coordinated the writing of the manuscript. AK coordinated the collaboration between Japan and Sweden. TK performed data analysis. TS supervised the study design and process. MB planned the Swedish part of the study including writing of manuscript.
